# Inverse Associations of Acetic Acid Intake with Carbohydrate, Sugar, and Saturated Fat Intakes Among Japanese Adults Aged 20 to 69 Years

**DOI:** 10.3390/nu18020318

**Published:** 2026-01-19

**Authors:** Risako Yamamoto-Wada, Joto Yoshimoto, Yoshino Kodaira, Kanako Deguchi, Yuto Aoki, Mikiya Kishi, Katsumi Iizuka

**Affiliations:** 1 Department of Clinical Nutrition, Fujita Health University, Toyoake 470-1192, Japan; risako.wada@fujita-hu.ac.jp (R.Y.-W.);; 2 Central Research Institute, Mizkan Holdings Co., Ltd., 2-6 Nakamura-Cho, Handa-Shi 475-8585, Japan; yoshimoto_jyoutou@mizkan.co.jp (J.Y.); yoshino_kodaira@mizkan.co.jp (Y.K.); yuto_aoki@mizkan.co.jp (Y.A.); mkishi1@mizkan.co.jp (M.K.); 3 Food and Nutrition Service Department, Fujita Health University Hospital, Toyoake 470-1192, Japan

**Keywords:** acetic acid, saturated fat, sugar, carbohydrate, food recording application, starch

## Abstract

**Background:** Acetic acid has been suggested to have health benefits. Our previous exploratory study linked acetic acid intake to higher protein and vitamin consumption, but relationships with age and sex remained unclear. **Objective:** This study examined associations between acetic acid intake, age, and sex, and explored nutrient correlates after adjusting for age, sex, and energy intake. **Methods:** Dietary data from 12,074 Japanese adults aged 20–69 years, collected via the Asken food-tracking app, were analyzed. Two-way ANOVA assessed effects of age, sex, and their interaction on acetic acid intake. Multiple linear regressions examined associations between acetic acid intake and nutrient intakes. Model 1 adjusted for age and sex; Model 2 additionally adjusted for total energy intake. **Results:** Participants included 3038 men (47.8 ± 11.9 y) and 9036 women (42.4 ± 11.8 y). Acetic acid intake was higher among men and older participants (sex: F = 11.0, *p* < 0.001; age: F = 9.1, *p* < 0.001). In Model 1, acetic acid intake correlated positively with most nutrients. After adjusting for energy (Model 2), negative associations were found with carbohydrates, sugars, starches, saturated fat, and butyric acid (all *p* < 0.05). **Conclusions:** Individuals with higher acetic acid intake tended to consume fewer carbohydrates and saturated fats, even at equivalent energy intake. These findings suggest that acetic acid-containing diets may reduce the intake of starches and saturated fatty acids, potentially contributing to obesity prevention.

## 1. Introduction

Vinegar, which contains acetic acid, was discovered in ancient times as a byproduct of alcohol and is currently used for preservation, medicinal purposes, and as a beverage [1,2]. In the Middle Ages, vinegar was produced using brewing techniques developed at the time and became widely used as a seasoning and preservative [1,2]. Since the early modern period, vinegar has been integrated into many regional food cultures, for the preparation of dishes such as sushi and for making pickles [1,2], while, from a commercial perspective, chemical analysis and industrialization have made mass production possible [1,2]. Worldwide, a variety of vinegars—such as balsamic vinegar, wine vinegar, apple cider vinegar, and rice vinegar, depending on local ingredients—are used as seasonings [1,2].

Acetic acid is recognized for its multifaceted effects, including the enhancement of sweetness and saltiness through its sourness, facilitation of color development, tenderization of meat and fish, promotion of calcium absorption, and antibacterial properties [1,2]. Moreover, acetic acid is widely regarded as a healthy food [3,4,5,6,7]. This compound has been reported to help prevent obesity and type 2 diabetes by regulating blood sugar levels and improving lipid metabolism, thereby reducing the risk of obesity and type 2 diabetes [3,4,5,6,7]. Specifically, it has been shown to impact appetite and satiety through delayed gastric emptying and increased feelings of fullness [8,9,10]. Furthermore, it has blood pressure-lowering and cholesterol-lowering effects [7,11,12].

Thus, although many pharmacological effects of acetic acid have been reported in recent years, there are very few reports on the relationship between acetic acid intake and disease. One reason for this is that acetic acid is a nutrient whose intake is difficult to measure. Another reason is the significant daily variation in acetic acid intake [13]. Food questionnaires rely on recent memory, whereas apps can record and analyze each meal as it is consumed, thus leading to differences in measurement methods [14]. Therefore, instead of using a questionnaire, we measured acetic acid intake using a food-recording app [13]. This app is similar to the paper-based diet recording method, but rather than being evaluated by a registered dietician, a computer calculates nutrient values on the basis of a menu database [13,14]. Using this method, we conducted an exploratory study with a small sample size and, for the first time, found a reported positive association between acetic acid intake and protein and vitamin intake [13]. However, owing to the small sample size, it was unclear how age and sex are related to acetic acid intake. Therefore, it was considered necessary to clarify the differences in acetic acid intake by age and sex in a larger population, as well as the relationship between acetic acid intake and the consumption of other nutrients.

Therefore, in this study, we aimed to clarify the associations between acetic acid intake and age, sex, energy intake, and the intake of various nutrients, such as protein, carbohydrates (dietary fiber, sugar, glucose, fructose, and sucrose), and lipids (saturated fat, monounsaturated fat, polyunsaturated fat, and butyric acid). First, using dietary data from 11,000 individuals, recorded via a food-recording app, we examined the effects of age and sex on acetic acid intake. Next, we analyzed the associations between acetic acid and other nutrients, adjusting for age, sex, and energy intake. Although acetic acid has long been known to have health benefits, such as preventing obesity and diabetes, sufficient evidence has not always been available—perhaps owing to the difficulty in measuring acetic acid levels. By using a meal tracking app to measure acetic acid, it will likely become possible to quantitatively evaluate the beneficial health effects of acetic acid, which has not been demonstrated until now.

## 2. Materials and Methods

### 2.1. Subjects

In this study, we purchased the following data from Asken as nonidentifiable anonymized data. Asken Inc. developed food recording apps, which are widely used in Japan. Dietary data were obtained from Asken users who provided informed consent for the use of their data for research purposes. For each individual, the acquired data included age, sex, energy intake, and nutritional values over multiple days. That is, we extracted data from individuals who had complete records of three meals per day for at least seven consecutive days during the period from Monday, 20 January 2025, to Monday, 10 February 2025. From these data, the extracted records were considered valid if they included entries for at least two out of three meals per day. The average recording period was 20.9 ± 2.6 days. Furthermore, the data were limited to those from individuals who were identified as male or female and whose ages were between 20 and 69 years. Since information about the participants was limited, no exclusion criteria were established. As a result, the study was conducted using data from 12,074 individuals aged 20 to 69. This study was conducted in accordance with the Declaration of Helsinki and was approved by the Ethics Committee of Fujita Health University (HM25-202, approval date 8 September 2025).

### 2.2. Data Collection from Food Recording Apps (Asken)

Food recording apps (Asken) are widely used in Japan. Energy and nutritional values were calculated from the dietary records following methods described in previous publications [13,14]. Briefly, the app automatically records and inputs the nutritional values linked to each item as entered (including snacks) by the user for each meal; thus, the daily nutritional intake is calculated automatically. Items recorded more than twice a day were considered valid, and the daily energy intake and nutritional values were calculated. The average energy intake and average nutrient intake measured over the specified period were defined as an individual’s energy intake and nutrient intake. Age, energy intake, and various nutritional values are presented as the means (SDs).

### 2.3. Statistical Analysis

#### 2.3.1. Two-Way ANOVA with Type III Sums of Squares (Type III ANOVA)

We performed two-way analysis of variance (ANOVA) with type III sums of squares to examine the effects of sex (male, female), age group (20–29, 30–39, 40–49, 50–59, and 60–69 years), and their interaction on each dietary and metabolic variable (energy, protein, carbohydrate, lipid, dietary fiber, total sugar, glucose, fructose, sucrose, acetic acid, butyric acid, saturated fat, monounsaturated fat, and polyunsaturated fat) calculated by the food recording app (Asken, Tokyo, Japan). This study was not conducted by controlling for each age group and gender, resulting in an unbalanced design with differing sample sizes in each group. Therefore, type III sums of squares ANOVA was applied to account for the unbalanced design.

Sex and age group were treated as categorical factors. For each dependent variable, a linear model was fitted that included sex, age group, and their interaction. Type III ANOVA was conducted via the Anova function in the car package (R Foundation for Statistical Computing). When a significant main effect was observed, post hoc comparisons between groups were carried out via Tukey’s honestly significant difference (HSD) test.

The F statistics and *p* values from the ANOVA were summarized, and the results of Tukey multiple comparisons (mean differences, confidence intervals, and adjusted *p* values) were tabulated. All analyses were performed in R (version 4.5.1, R Foundation for Statistical Computing). *p* < 0.05 was considered statistically significant.

#### 2.3.2. Statistical Visualization

For each outcome variable, we fitted a two-way analysis of variance model with sex, age group, and their interaction (age × sex) as fixed effects. From these models, we obtained estimated marginal means (EMMs) for the age group within each sex and their 95% confidence intervals via the estimated marginal means (EMMs) package in R. EMMs were plotted with points and error bars and connected by lines to display simple effects of age across sex. One figure was generated per outcome and exported in PNG format (7 × 5 in, 300 dpi). In addition to EMMs, analyses were conducted in R (version 4.5.1) via ggplot2 (4.0.0) and dplyr (2.5.0).

#### 2.3.3. Linear Regression Models Adjusted for Sex and Age (Model 1) and Sex, Age, and Energy Intake (Model 2)

Associations between acetic acid intake and individual nutrients were examined using multiple linear regression analyses. The independent variable was mean daily acetic acid intake (acetic_acid_mean), and each nutrient was analyzed separately as a dependent variable. Two models were constructed: Model 1 adjusted for age and sex, and Model 2 additionally adjusted for total energy intake. All continuous variables were standardized to z-scores, and standardized regression coefficients (β) with 95% confidence intervals (CIs) and *p* values were obtained. Partial correlation coefficients (r) were also calculated using the ppcor package in R to quantify the strength of each association after covariate adjustment. Ninety-five percent CIs for r were derived via Fisher’s z transformation. Both standardized regression coefficients (β) and partial correlation coefficients (partial r) were reported as effect-size measures. Standardized β represents the relative contribution of each predictor within the regression model, whereas partial r reflects the strength of association between two variables after controlling for covariates. Effect sizes were interpreted as small (0.10–0.29), medium (0.30–0.49), or large (≥0.50). Estimates and CIs were reported with two significant digits, and *p* < 0.001 was considered significant. Results were visualized as dual-panel forest plots comparing standardized β and partial r across Model 1 and Model 2 using ggplot2 in R. The results were compiled for all the predictors; plots were generated to display βstd with 95%CIs for both models, with the y-axis order fixed by the absolute magnitude of the Model 1 estimates. Figures were exported as PNG files, and a companion table including βstd, 95%CIs, and *p* values was generated. Analyses were conducted in R (version 4.5.1) via readr (2.1.5), dplyr (2.5.0), broom (1.0.9), ggplot2 (4.0.0), ppcor (1.1), and tidyr (1.3.1).

## 3. Results

### 3.1. Demographic Characteristics of the Study Participants

On the basis of nutrient intake data derived from the daily dietary records of the users of the food recording app (Asken), we aimed to clarify the associations between acetic acid intake and age, sex, and the interaction between age and sex. Next, we aimed to clarify the associations between acetic acid intake and the intake of various nutrients.

With respect to the background of the study participants, the data for 12,074 individuals were included in the analysis, comprising 3038 men and 9036 women aged 20–69 years. The mean ± SD ages of males and females were 47.8 ± 11.9 and 42.38 ± 11.8 years, respectively. Energy intake was roughly stable across age groups in men at about 2000 kcal/day and in women at about 1500–1600 kcal/day (Table 1). Acetic acid intake ranged from about 0.21 to 0.27 kcal/day in men from their 20 s to 60 s, and from about 0.16 to 0.22 kcal/day in women in the same age range (Table 1). Table 1 summarizes the observed values for energy and nutrient intake by age group and sex.

### 3.2. Associations Between Nutrients and Age, Sex, and Their Interaction

#### 3.2.1. Associations Between Energy, Protein, and Carbohydrate and Age, Sex, and Their Interaction

Next, we examined the effects of age and sex on nutrient intake (including acetic acid intake). For each dietary variable, we conducted two-way ANOVA (type III), including age group, sex, and their interaction (age × sex) as factors (Table 2).

**Figure 1:**
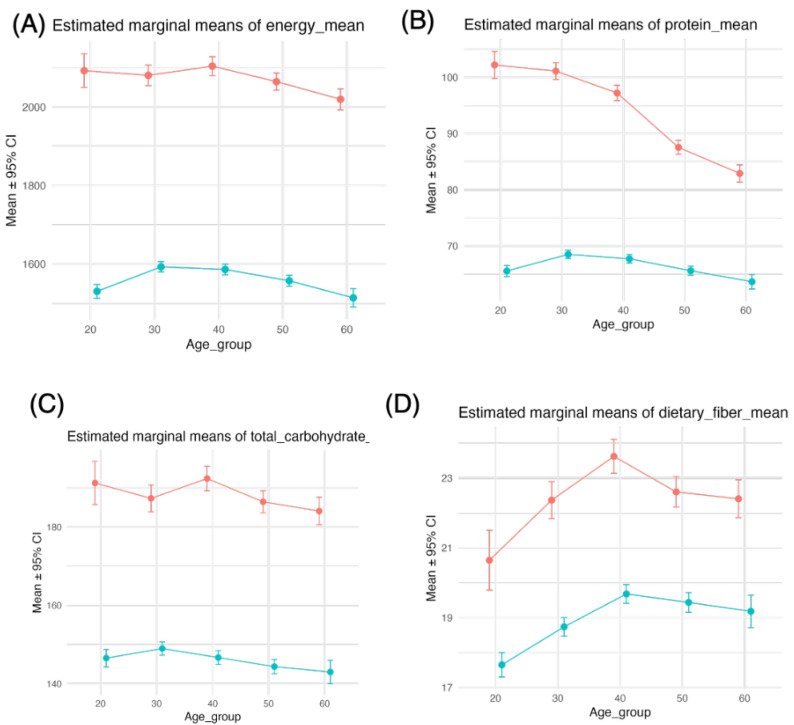

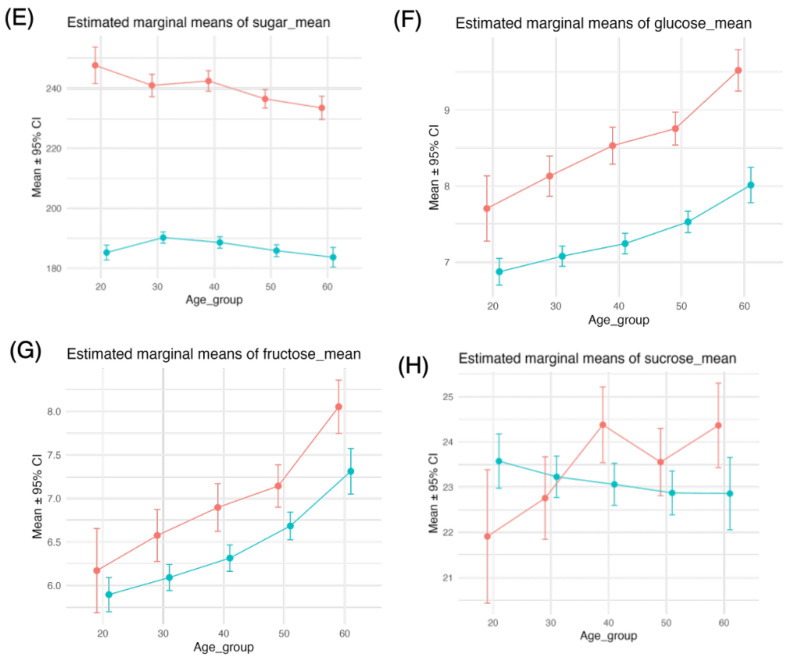
Two-way ANOVA (type III) for energy, protein, carbohydrate, dietary fiber, sugar, glucose, fructose, and sucrose intakes. We performed two-way analysis of variance (ANOVA) with type III sums of squares to examine the effects of sex group, age group, and their interaction on each dietary and metabolic variable (energy (**A**), protein (**B**), carbohydrate (**C**), dietary fiber (**D**), total sugar (**E**), glucose (**F**), fructose (**G**), and sucrose (**H**)). From these models, we obtained estimated marginal means (EMMs) for the age group within each sex stratum and their 95% confidence intervals via the emmeans package. EMMs were plotted with points and error bars and connected by lines to display simple effects of age across sex groups. Red and blue indicate males and females, respectively.

Energy intake gradually decreased from the thirties age group onward, and in every age group, energy intake was greater among men (Figure 1A). Two-way ANOVA (type III) revealed significant main effects of sex (F(1, 12,074) = 574.0, *p* < 0.001) and age group (F(4, 12,074) = 5.7, *p* < 0.001) on energy intake, but no significant sex × age interaction was detected (F(4, 12,074) = 1.9, *p* = 0.11) (Table 2, Figure 1A). Protein and carbohydrate intake decreased with age, and the sex difference decreased with increasing age (Table 2, Figure 1B,C). The results of the ANOVA revealed that for protein and carbohydrate intake, all the main and interaction effects were significant (protein intake–sex: F(1, 12,074) = 761.0, *p* < 0.001; age: F(4, 12,074) = 113.0, *p* < 0.001; sex × age: F(4, 12,074) = 52.0, *p* < 0.001; carbohydrate intake–sex: F(1, 12,074) = 344, *p* < 0.001; age: F(4, 12,074) = 5.2, *p* < 0.001; sex × age: F(4, 12,074) = 2.7, *p* = 0.029) (Table 2).

For both sexes, dietary fiber intake increased in individuals until their forties but plateaued thereafter, while similar sex differences were observed across age groups (Figure 1D). Moreover, according to the ANOVA results, the main effects were significant (sex: F(1, 12,074) = 40, *p* < 0.001; age: F(4, 12,074) = 9.5, *p* < 0.001), but the interaction effect was not significant (sex × age: F(4, 12,074) = 1.5, *p* = 0.21) (Table 2). The findings for sugars (which include carbohydrates without dietary fiber) were similar to those for carbohydrates (Figure 1E, Table 2). With respect to sugar intake, all the main and interaction effects were significant (sex: F(1, 12,064) = 350, *p* < 0.001; age: F(4, 12,074) = 5.8, *p* < 0.001; sex × age: F(4, 12,074) = 3.0, *p* = 0.017) (Table 2). Glucose and fructose intake increased monotonically across age groups, with comparable sex differences in each age group (Figure 1F,G). For glucose intake, only the main effects were significant (sex: F(1, 12,074) = 12, *p* < 0.001; age: F(4, 12,074) = 19, *p* < 0.001) (Table 2). In contrast, only sex had a significant effect on fructose intake (F(1, 12,074) = 16, *p* < 0.001) (Table 2).

Sucrose intake increased with age in men but remained stable in women, resulting in a widening sex gap with increasing age (Figure 1H). All the main and interaction effects were significant (sex: F(1, 12,074) = 4.2, *p* = 0.040; age: F(4, 12,074) = 3.6, *p* = 0.006; sex × age: F(4, 12,074) = 4.0, *p* = 0.003) (Table 2).

Thus, the intake of glucose and fructose was greater among men across all age groups and increased with age.

#### 3.2.2. Associations Between Lipids and Age, Sex, and Their Interaction

**Figure 2:**
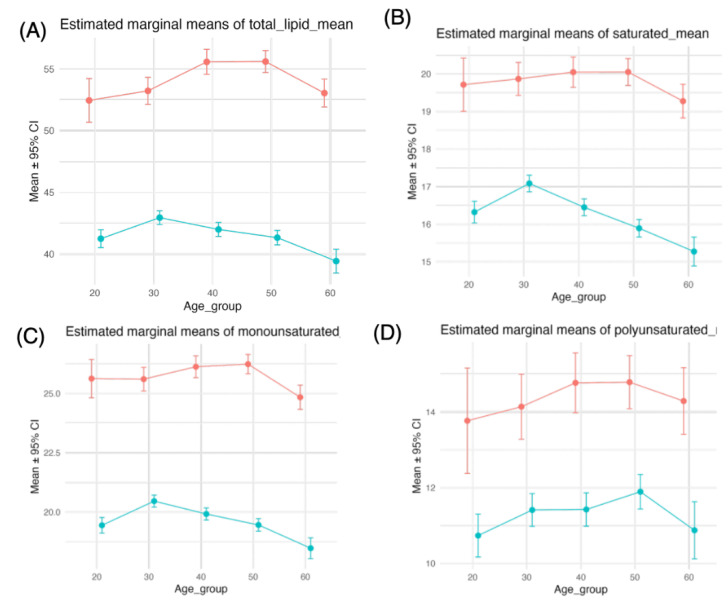
Two-way ANOVA (type III) for lipid, saturated fat, monounsaturated fat, and polyunsaturated fat intakes. We performed two-way analysis of variance (ANOVA) with type III sums of squares to examine the effects of sex group, age group, and their interaction on each dietary and metabolic variable (lipid (**A**), saturated fat (**B**), monounsaturated fat (**C**), and polyunsaturated fat (**D**)). From these models, we obtained estimated marginal means (EMMs) for the age group within each sex stratum and their 95% confidence intervals via the emmeans package. EMMs were plotted with points and error bars and connected by lines to display simple effects of age across sex groups. Red and blue indicate males and females, respectively.

Lipid intake decreased with age in women, resulting in widening sex differences across the age groups (Figure 2A, Table 2). All the main and interaction effects were significant (sex: F(1, 12,074) = 194, *p* < 0.001; age: F(4, 12,074) = 4.2, *p* = 0.002; sex × age: F(4, 12,074) = 4.6, *p* = 0.001) (Table 2).

Notably, the intake of saturated fatty acids and monounsaturated fatty acids decreased in women older than 30 years but not in men, resulting in more pronounced sex differences with age (Figure 2B,C). For saturated fat intake, only sex and interaction effects were significant (sex: F(1, 12,074) = 76, *p* < 0.001; age: F(4, 12,074) = 2.1, *p* = 0.073; sex × age: F(4, 12,074) = 4.8, *p* < 0.001) (Table 2, Figure 2B), whereas for monounsaturated fat intake, all the main and interaction effects were significant (sex: F(1, 12,074) = 193, *p* < 0.001; age: F(4, 12,074) = 5.1, *p* < 0.001; sex × age: F(4, 12,074) = 4.8, *p* < 0.001) (Table 2, Figure 2C). PUFA intake was greater for men than for women, and the sex differences were relatively consistent across age groups (Figure 2D). For polyunsaturated fat intake, only the sex effect was significant (sex: F(1, 12,074) = 16, *p* < 0.001) (Table 2, Figure 2D).

#### 3.2.3. Associations Between Acetic Acid and Age, Sex, and Their Interaction

**Figure 3:**
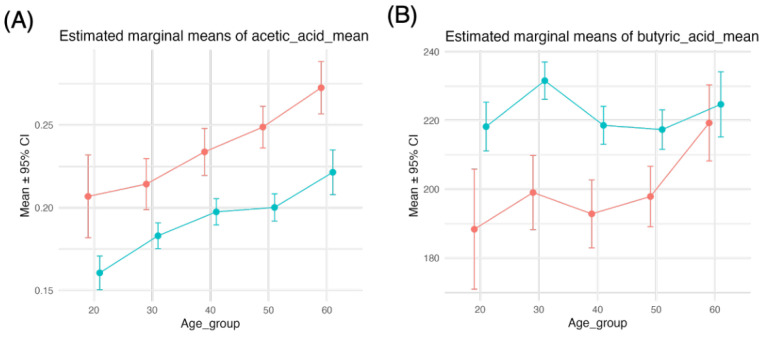
Two-way ANOVA (type III) for acetic acid and butyric acid intakes. We performed two-way analysis of variance (ANOVA) with type III sums of squares to examine the effects of sex group, age group, and their interaction on each dietary and metabolic variable (acetic acid (**A**) and butyric acid (**B**)). From these models, we obtained estimated marginal means (EMMs) for the age group within each sex stratum and their 95% confidence intervals via the emmeans package. EMMs were plotted with points and error bars and connected by lines to display simple effects of age across sex groups. Red and blue indicate males and females, respectively.

Acetic acid intake increased monotonically with age, and comparable sex differences were observed across age groups (Table 2, Figure 3A). For acetic acid intake, only the main effects were significant (sex: F(1, 12,074) = 11, *p* < 0.001; age: F(4, 12,074) = 9.1, *p* < 0.001; sex × age: F(4, 12,074) = 0.88, *p* = 0.47) (Table 2). Butyric acid intake increased with age only in men, leading to a narrowing sex difference (Figure 3B). For butyric acid intake, only the main effects were significant (sex: F(1, 12,074) = 9.6, *p* = 0.002; age: F(4, 12,074) = 3.9, *p* = 0.004; sex × age: F(4, 12,074) = 2.3, *p* = 0.057) (Table 2, Figure 3B).

Thus, the intake of acetic acid was greater among men across all age groups, increased with age, and showed a pattern similar to that of glucose and fructose intake; however, no interaction between age and sex was observed.

### 3.3. Associations Between Acetic Acid Intake and Each Nutrient Adjusted for Age, Sex, and Energy Intake

**Figure 4:**
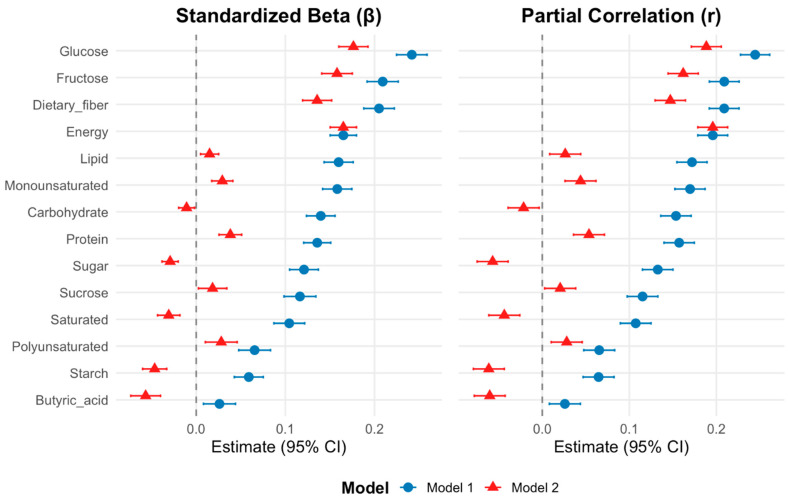
Associations between acetic acid and nutrient intake (standardized regression coefficients ± 95%CI; partial correlation coefficients(r) with 95%CI). Multivariable regression models were constructed with acetic acid as the independent variable and the intake of different nutrients as the dependent variable. Model 1 was adjusted for age and sex, and Model 2 was additionally adjusted for energy intake. Rightward estimates indicate positive associations, whereas leftward estimates indicate inverse associations. Red and blue indicate Model 1 and Model 2, respectively. Acetic acid intake was negatively associated with carbohydrates, sugar, saturated fatty acids, starch, and butyric acid only when adjusted for age, sex, and energy.

Finally, we sought to assess the associations between acetic acid intake and the intake of other nutrients after adjusting for sex, age, and energy intake (Figure 4 and Table 3).

Multivariable analyses were conducted via two models (Model 1: adjusted for age and sex; Model 2: adjusted for age, sex, and energy intake), with acetic acid as the dependent variable and different nutrients as independent variables (Figure 4 and Table 3). In multiple linear regression analyses, standardized regression coefficients (β) and partial correlation coefficients (partial r) were calculated to evaluate the strength of associations between acetic acid intake and each nutrient, adjusting for age and sex (Model 1) and additionally for energy intake (Model 2).

In Model 1, acetic acid intake was positively associated with the intake of glucose (g/day) (β Standard [95%CI]:0.24 [0.22–0.26], <0.001; partial r [95%CI]: 0.24 [0.23–0.26], <0.001), dietary fiber (g/day) (β Standard [95%CI]:0.21 [0.19–0.22], <0.001; partial r [95%CI]:0.21 [0.19–0.23], <0.001), fructose (g/day) (β Standard [95%CI]:0.21 [0.19–0.23], <0.001; partial r [95%CI]: 0.21 [0.19–0.23], <0.001). These changes were also observed in model 2, and were not affected by energy intake (glucose (β Standard [95%CI]:0.18 [0.16–0.19], <0.001; partial r [95%CI]: 0.19 [0.17–0.21], <0.001), dietary fiber (β Standard [95%CI]: 0.14 [0.12–0.15], <0.001; partial r [95%CI]: 0.15 [0.13–0.16], <0.001), and fructose (β Standard [95%CI]: 0.16 [0.14–0.18], <0.001; partial r [95%CI]: 0.16 [0.14–0.18], <0.001).

In contrast, in Model 2, acetic acid intake was inversely associated with the intakes of carbohydrate, total sugar, saturated fatty acid, starch, and butyric acid (carbohydrates: β Std [95%CI]; −0.01 [−0.02–−1.82 × 10^−3^], *p* = 0.018; partial r [95%CI]:−0.02 [−0.04–−3.64 × 10^−3^], *p* = 0.018; total sugars: β Std [95%CI]; −0.03 [0.04–0.02], *p* < 0.001; partial r [95%CI]: −0.06 [0.07–0.04], *p* < 0.001; saturated fatty acids: β Std [95%CI]; −0.03 [0.04–0.02], *p* < 0.001; partial r [95%CI]: −0.04 [0.06–0.03], *p* < 0.001; starches: β Std [95%CI]; −0.05 [0.06–0.03], <0.001; partial r [95%CI]: −0.06 [0.08–0.04], <0.001; and butyric acid: β Std [95%CI]; −0.06 [0.07–0.04], *p* < 0.001; partial r [95%CI]: −0.06 [0.08–0.04], *p* < 0.001) (Figure 4 and Table 3).

Thus, most nutrients were significantly associated with acetic acid intake (*p* < 0.05); however, both the standardized β coefficients and partial correlation coefficients were small (0.1–0.3), indicating weak associations. Importantly, acetic acid intake was negatively associated with carbohydrate, sugar, starch, saturated fatty acid, and butyric acid intake in Model 2, indicating that positive association observed in Model 1 was largely confounded by total energy intake.

## 4. Discussion

Acetic acid is considered beneficial for the body; however, the types of foods associated with its intake have not been thoroughly investigated. In our previous exploratory study, involving a food recording app, we reported positive correlations between acetic acid intake and protein and vitamin intake. In this study, which involved approximately 10,000 people, we clarified the differences in acetic acid intake according to age and sex. Furthermore, we examined the relationships between the intake of acetic acid and other nutrients after adjusting not only for age and sex but also for energy intake. The results revealed that acetic acid intake was greater in men across all age groups and increased steadily with age; a similar pattern was observed for glucose and fructose intake. Next, we investigated the relationships between acetic acid intake and the intake of various nutrients. When we performed linear regression analyses adjusted for age and sex, we found a positive correlation between acetic acid intake and most nutrients, such as dietary fiber. However, when we further adjusted for energy intake, we observed negative correlations with the intake of carbohydrates, total sugars, starch, saturated fatty acids, and butyric acid. Excessive intake of carbohydrates and saturated fatty acids can lead to obesity, arteriosclerosis, and diabetes [15,16]. Therefore, if acetic acid intake is high while carbohydrate and saturated fatty acid intake are low, dishes prepared with vinegar (which includes acetic acid) may help prevent obesity and diabetes.

Acetic acid intake was greater in men and increased with age in both sexes. The age-related increase may be related to greater dietary diversity in older age groups. Our study revealed that dietary diversity increased with age. Seasonings containing acetic acid are often used in side dishes, supporting these findings. Additionally, it is possible that taste preferences change with age. Many elderly people experience reduced sensitivity to taste and tend to find it harder to perceive sour flavors, which may lead to increased consumption of foods with a strong sour taste [17]. Furthermore, from middle age onward, individuals may become more conscious of weight gain and the risks of lifestyle-related diseases, leading to a tendency to avoid greasy foods and prefer lighter dishes [18,19]. Thus, changes in taste perception and health awareness with age may play a role in increased acetic acid intake. However, the reasons for these sex differences remain unclear. A cross-sectional survey in the United States (Beaver Dam Offspring Study, *n* = 2374) revealed that women are more sensitive to sourness than men [20]. Additionally, a study involving 1020 Europeans revealed that women who perceive sourness more intensely also have a greater preference for sour tastes [21]. Although the absolute intake of acetic acid is higher in men than in women across all age groups, this gender difference disappears when adjusted for total energy intake. Therefore, this does not contradict the finding that women tend to prefer sour flavors. Thus, acetic acid intake increased with age and differed by sex, possibly due to age-related changes in dietary preferences and taste perception. Future studies are needed to elucidate the relationship between food preferences and acetic acid intake.

The behavior of glucose and fructose was similar to that of acetic acid. Vinegars such as black rice vinegar, citrus vinegar, and white rice vinegar often contain glucose and fructose. While the concentration of these sugars varies depending on the type of vinegar, the sugar content is often less than 1%, compared with 4–5% acetic acid. Therefore, the similarity in behavior between vinegar and glucose or fructose may not be due to the vinegar itself. On the other hand, vinegar is also used as an ingredient in seasonings such as dressings, Worcestershire sauce, and ketchup, where sugars are frequently added at the same time. High-fructose corn syrup is less expensive than sugar and is often used in dressings, Worcestershire sauce, and ketchup. It may also be added to beverages containing vinegar and to pickles. Thus, the similarity in the behavior of acetic acid, glucose, and fructose observed in this study was presumed to result from the increased consumption of vinegar-containing seasonings, beverages, and processed foods. Since sugars such as glucose and fructose can cause obesity and tooth decay, excessive use of dressings and mayonnaise should be avoided.

After adjusting for energy intake, age and sex, we found a negative relationship between acetic acid intake and the intake of carbohydrates, starches, and saturated fatty acids. These findings are consistent with reports that acetic acid intake is useful for preventing obesity [3,4,5,6,7], since carbohydrates and starches are used to synthesize fatty acids in the liver and adipose tissue, and saturated fatty acids are also stored in fat tissue. Although the causal relationship remains unclear, higher acetic acid intake appears to be associated with lower carbohydrate, starch, and saturated fatty acid intake, suggesting that a diet including acetic acid may be more effective in preventing lifestyle-related diseases. Moreover, a previous study revealed that the degree of starch digestion in food was negatively correlated with acetic acid concentration [22,23,24,25,26]. For example, vinegars with higher contents of various organic acids have strong potential against digestive enzymes such as α-amylase and α-glucosidase. Moreover, in normoglycemic subjects, it was revealed that 20 mL white vinegar (5% acetic acid) as a salad dressing ingredient reduced the glycemic response to a mixed meal (lettuce salad and white bread containing 50 g carbohydrate) by over 30% (*p* < 0.05) [25]. Moreover, by prolonging gastric emptying, the presence of vinegar significantly reduced the postprandial glucose and insulin responses to a starchy meal [26]. The habit of drinking a lot of vinegar may help suppress the rise in blood sugar not only by inhibiting the action of digestive enzymes and slowing down stomach peristalsis, but also by reducing the intake of carbohydrates themselves.

The relationship between saturated fatty acids and acetic acid is also intriguing. In animal studies, the administration of acetic acid has been shown to reduce cholesterol and neutral fat levels, with reported decreases in hepatic fatty acid synthase and enhanced fatty acid oxidation [7,27]. In humans, meta-analysis data on the effects of acetic acid administration on neutral fat have been reported [28]. Dietary acetic acid supplementation resulted in significant reductions in TAG concentrations in overweight and obese but otherwise healthy individuals (mean difference [MD] = −20.51 mg/dL [95% confidence intervals = −32.98, −8.04], *p* = 0.001) and people with type 2 diabetes (MD = −7.37 mg/dL [−10.15, −4.59], *p* < 0.001). The reduction in neutral fat caused by acetic acid intake may be partly related to a decrease in the intake of saturated fatty acids. In future studies, it will be necessary to conduct a detailed dietary survey during trials involving acetic acid administration, evaluate the energy and nutrient intake during the study period, and confirm the effects of acetic acid on diet.

With respect to the study’s limitations, we lack data on BMI and therefore cannot exclude the influence of body size on the observed effects. Since clinical studies have reported weight loss effects from acetic acid intake, further clarification of the relationship between body size and acetic acid intake is necessary. Second, as this was an exploratory study, we did not predetermine the number of participants. However, since we found significant associations between the intake of nearly all nutrients—including, for the first time, dietary fiber—and not just protein and vitamin intake, as reported in our previous study, the sample size can be considered sufficient. Since this study lacked information on BMI and underlying health conditions, future large-scale studies incorporating BMI, as well as age and sex, will be necessary to obtain a more comprehensive understanding.

## 5. Conclusions

Through this study, we revealed that acetic acid intake is affected by age and sex and is negatively associated with the intake of starches, carbohydrates, and saturated fatty acids. Future studies should seek to clarify the relationships among acetic acid intake, nutrient intake, and body composition in actual daily dietary records after adjusting for age and sex. Moreover, as this was not analyzed in this study, we plan to clarify the relationship between food preferences and acetic acid intake. It may be possible to estimate acetic acid intake on the basis of individual dietary preferences. Using a food recording application to estimate acetic acid intake, future research should aim to elucidate the associations of acetic acid intake not only with the intake of other nutrients but also with nutritional status and disease risk.

## Figures and Tables

**Table 1:** Background of the individuals in this study.

Sex	Male	Male	Male	Male	Male	Female	Female	Female	Female	Female
Age group	20 s	30 s	40 s	50 s	60 s	20 s	30 s	40 s	50 s	60 s
n	231	605	718	909	575	1394	2398	2314	2144	786
Energy (kcal)	2092.8 (400.1)	2080.8 (410.5)	2104.3 (438.5)	2064.9 (387.8)	2019.8 (354.0)	1530.5 (329.4)	1593.0 (333.4)	1586.3 (299.5)	1557.5 (276.2)	1514.1 (234.4)
Protein (g)	102.2 (29.7)	101.1 (27.9)	97.2 (28.9)	87.5 (23.2)	82.9 (19.9)	65.5 (16.2)	68.5 (16.4)	67.7 (16.1)	65.6 (15.1)	63.6 (12.9)
Carbohydrate (g)	268.4 (63.2)	263.4 (64.4)	266.1 (65.4)	259.1 (58.7)	255.9 (56.2)	202.9 (47.9)	209.0 (47.9)	208.3 (44.8)	205.3 (43.1)	202.9 (35.6)
Dietary fiber (g)	20.6 (6.1)	22.4 (7.8)	23.6 (8.5)	22.6 (7.2)	22.4 (7.1)	17.7 (6.4)	18.7 (6.2)	19.7 (6.7)	19.4 (6.2)	19.2 (5.7)
Sugar (g)	247.7 (60.4)	241.0 (62.2)	242.5 (61.4)	236.5 (56.0)	233.5 (53.0)	185.3 (45.1)	190.3 (45.4)	188.6 (42.3)	185.9 (40.6)	183.7 (33.6)
Glucose (g)	7.7 (3.9)	8.1 (4.1)	8.5 (3.8)	8.8 (3.6)	9.5 (4.1)	6.9 (3.0)	7.1 (3.3)	7.2 (3.0)	7.5 (3.1)	8.0 (3.3)
Fructose (g)	6.2 (4.2)	6.6 (4.6)	6.9 (4.2)	7.1 (4.2)	8.1 (4.6)	5.9 (3.3)	6.1 (3.5)	6.3 (3.6)	6.7 (3.5)	7.3 (3.6)
Sucrose (g)	21.9 (12.3)	22.8 (14.5)	24.4 (13.6)	23.6 (12.8)	24.4 (12.7)	23.6 (11.9)	23.2 (11.0)	23.1 (10.5)	22.9 (10.4)	22.9 (9.0)
Lipid (g)	70.1 (18.1)	70.7 (19.2)	72.2 (20.4)	72.1 (17.4)	69.2 (16.2)	54.6 (15.7)	57.5 (16.2)	56.3 (14.4)	55.0 (13.4)	52.6 (11.3)
Saturated fat (g)	19.7 (6.1)	19.9 (7.3)	20.1 (6.8)	20.1 (5.9)	19.3 (5.1)	16.3 (5.5)	17.1 (5.9)	16.5 (5.0)	15.9 (4.7)	15.3 (4.1)
Monounsaturated fat (g)	25.6 (7.3)	25.6 (7.7)	26.1 (8.4)	26.2 (7.1)	24.8 (6.7)	19.4 (6.2)	20.5 (6.2)	19.9 (5.6)	19.5 (5.8)	18.5 (4.4)
Polyunsaturated fat (g)	13.8 (3.6)	14.1 (3.8)	14.8 (4.2)	14.8 (3.8)	14.3 (4.0)	10.7 (3.2)	11.4 (3.1)	11.4 (2.9)	11.9 (24.6)	10.9 (2.6)
Acetic acid (g)	0.21 (0.20)	0.21 (0.20)	0.23 (0.23)	0.25 (0.22)	0.27 (0.26)	0.16 (0.14)	0.18 (0.17)	0.20 (0.19)	0.20 (0.19)	0.22 (0.22)
Butyric acid (mg)	188.4 (127.4)	199.1 (199.0)	192.9 (139.7)	197.9 (136.4)	219.3 (137.0)	218.2 (126.0)	231.6 (141.3)	218.6 (126.7)	217.4 (122.8)	224.7 (125.7)

**Table 2:** Two-way ANOVA (type III) for dietary/nutrient intakes factors: sex, age, and sex × age interaction.

Outcome	Sex (F(1, 12,074), *p*)	Age (F(4, 12,074), *p*)	Sex × Age ((4, 12,074), *p*)
**Energy (kcal/day)**	**574, <0.001**	**5.7, <0.001**	1.9, 0.11
**Protein (g/day)**	**761, <0.001**	**113, <0.001**	**52, <0.001**
**Carbohydrate (g/day)**	**344, <0.001**	**5.2, <0.001**	**2.7, 0.029**
**Dietary fiber (g/day)**	**40, <0.001**	**9.5, <0.001**	1.5, 0.21
**Total Sugar (g/day)**	**350, <0.001**	**5.8, <0.001**	**3.0, 0.017**
**Glucose (g/day)**	**12, <0.001**	**19, <0.001**	1.6, 0.16
**Fructose (g/day)**	1.1, 0.30	**16, <0.001**	0.59, 0.67
**Sucrose (g/day)**	**4.2, 0.040**	**3.6, 0.006**	**4.0, 0.003**
**Lipid (g/day)**	**194, <0.001**	**4.2, 0.002**	**4.6, 0.001**
**Saturated fat (g/day)**	**76, <0.001**	2.1, 0.073	**4.8, <0.001**
**Monounsaturated fat (g/day)**	**193, <0.001**	**5.1, <0.001**	**4.8, <0.001**
**Polyunsaturated fat (g/day)**	**16, <0.001**	0.76, 0.55	0.34, 0.85
**Acetic acid (g/day)**	**11, <0.001**	**9.1, <0.001**	0.88, 0.47
**Butyric acid (mg/day)**	**9.6, 0.002**	**3.9, 0.004**	2.3, 0.057

Bold indicates significant (*p* < 0.05).

**Table 3:** Associations of nutrient intake with acetic acid levels (standardized β with 95%CI, partial correlation coefficients(r) with 95%CI), under two adjustment models.

Outcome	Model 1: β [95%CI], *p*	Model 1: r [95%CI], *p*	Model 2: β [95%CI], *p*	Model 2: r [95%CI], *p*
glucose_mean	0.24 [0.22–0.26], <0.001	0.24 [0.23–0.26], <0.001	0.18 [0.16–0.19], <0.001	0.19 [0.17–0.21], <0.001
fructose_mean	0.21 [0.19–0.23], <0.001	0.21 [0.19–0.23], <0.001	0.16 [0.14–0.18], <0.001	0.16 [0.14–0.18], <0.001
dietary_fiber_mean	0.21 [0.19–0.22], <0.001	0.21 [0.19–0.23], <0.001	0.14 [0.12–0.15], <0.001	0.15 [0.13–0.16], <0.001
energy_mean	0.17 [0.15–0.18], <0.001	0.2 [0.18–0.21], <0.001	0.17 [0.15–0.18], <0.001	0.2 [0.18–0.21], <0.001
lipid_mean	0.16 [0.14–0.18], <0.001	0.17 [0.15–0.19], <0.001	0.01 [+4.86 × 10^−3^–+0.03], *p* = 0.004	0.03 [0.01–0.04], *p* = 0.004
monounsaturated_mean	0.16 [0.14–0.17], <0.001	0.17 [0.15–0.19], <0.001	0.03 [0.02–0.04], <0.001	0.04 [0.03–0.06], <0.001
protein_mean	0.14 [0.12–0.15], <0.001	0.16 [0.14–0.17], <0.001	0.04 [0.03–0.05], <0.001	0.05 [0.04–0.07], <0.001
carbohydrate_mean	0.14 [0.12–0.16], <0.001	0.15 [0.14–0.17], <0.001	−0.01 [−0.02–−1.82 × 10^−3^], *p* = 0.018	−0.02 [−0.04–−3.64 × 10^−3^], *p* = 0.018
sugar_mean	0.12 [0.1–0.14], <0.001	0.13 [0.12–0.15], <0.001	−0.03 [−0.04–−0.02], <0.001	−0.06 [−0.07–−0.04], <0.001
sucrose_mean	0.12 [0.1–0.13], <0.001	0.12 [0.1–0.13], <0.001	0.02 [+2.45 × 10^−3^–+0.03], *p* = 0.024	0.02 [+2.75 × 10^−3^–+0.04], *p* = 0.024
saturated_mean	0.1 [0.09–0.12], <0.001	0.11 [0.09–0.12], <0.001	−0.03 [−0.04–−0.02], <0.001	−0.04 [−0.06–−0.03], <0.001
polyunsaturated_mean	0.07 [0.05–0.08], <0.001	0.07 [0.05–0.08], <0.001	0.03 [0.01–0.05], *p* = 0.002	0.03 [0.01–0.05], *p* = 0.002
starch_mean	0.06 [0.04–0.07], <0.001	0.07 [0.05–0.08], <0.001	−0.05 [−0.06–−0.03], <0.001	−0.06 [−0.08–−0.04], <0.001
butyric_acid_mean	0.03 [0.01–0.04], *p* = 0.004	0.03 [0.01–0.04], *p* = 0.004	−0.06 [−0.07–−0.04], <0.001	−0.06 [−0.08–−0.04], <0.001

The independent variable was mean daily acetic acid intake (acetic_acid_mean), and each nutrient was analyzed separately as a dependent variable. Two models were constructed: Model 1 adjusted for age and sex, and Model 2 additionally adjusted for total energy intake. All continuous variables were standardized to z-scores, and standardized regression coefficients (β), and 95% confidence intervals (CIs) and *p* values were obtained. Partial correlation coefficients (r) were also calculated using the ppcor package in R to quantify the strength of each association after covariate adjustment. Ninety-five percent CIs for r were derived via Fisher’s z transformation. Both standardized regression coefficients (β) and partial correlation coefficients (partial r) were reported as effect size measures. Acetic acid intake was negatively associated with carbohydrates, sugar, saturated fatty acids, starch, and butyric acid only when adjusted for age, sex, and energy. Red letter indicates cases where β Std for Model 1 is positive and β Std for Model 2 is negative.

## Data Availability

The datasets presented in this article are not readily available because the data are part of an ongoing study.
